# Exploring Motivation to Participate in Cognitive Training Across Underrepresented Groups in Clinical Research

**DOI:** 10.1093/geroni/igaf122.299

**Published:** 2025-12-31

**Authors:** Lizbeth Vera Murillo, Maria Vander Meulen, Angel Collie, Stacey Alvarez-Alvarado, Kat Barrett, Yuting Yang, Jennifer O’Brien

**Affiliations:** Duke University School of Medicine, Durham, North Carolina, United States; University of Florida College of Medicine - Jacksonville, Jacksonville, Florida, United States; The Pennsylvania State University, University Park, Pennsylvania, United States; University of Florida College of Medicine - Jacksonville, Jacksonville, Florida, United States; Clemson University, Clemson, South Carolina, United States; Health Informatics Institute, Tampa, Florida, United States; University of South Florida, Tampa, Florida, United States

## Abstract

Challenges with participant recruitment and lack of sample diversity are common problems in clinical research. The Alzheimer’s Association highlights the need for more heterogeneous samples in clinical research; underrepresented groups in clinical research can be categorized by demographic characteristics, including race/ethnicity, gender, and participant age. In particular, more effective recruitment for Latinos/Hispanics, Blacks/African Americans, males, and old-old adults (≥75 years) is needed. The goal of the current study was to explore motivations for participating in an Alzheimer’s disease and related dementias (ADRD) prevention trial and to examine whether motivations differed by demographic characteristics associated with underrepresented groups in clinical research. Cognitively healthy older adults (N = 5,721, Mage= 71.5 + 5.0) enrolled in the Preventing Alzheimer’s with Cognitive Training (PACT) trial completed open-ended questions assessing motivations for participation. Thematic analyses were conducted to identify motivational topics. Demographic subgroup differences were analyzed using chi-square and logistic regression analyses. Concern about brain health was the most common motivational theme, followed by direct experience with ADRD and altruism. Motivations differed by gender, race, age, and education. Results suggest that focusing on brain health concerns may be effective for recruitment messaging in ADRD prevention trials. However, demographic differences in motivations suggest recruitment materials and messaging could be tailored to align with specific motivational factors among subgroups.

